# Posttraumatic Stress in Survivors 1 Month to 19 Years after an Airliner Emergency Landing

**DOI:** 10.1371/journal.pone.0119732

**Published:** 2015-03-03

**Authors:** Filip K. Arnberg, Per-Olof Michel, Tom Lundin

**Affiliations:** 1 National Centre for Disaster Psychiatry, Department of Neuroscience, Uppsala University, Sweden; 2 Stress Research Institute, Stockholm University, Stockholm, Sweden; Univ of Toledo, UNITED STATES

## Abstract

Posttraumatic stress (PTS) is common in survivors from life-threatening events. Little is known, however, about the course of PTS after life threat in the absence of collateral stressors (e.g., bereavement, social stigma, property loss) and there is a scarcity of studies about PTS in the long term. This study assessed the short- and long-term course of PTS, and the influence of gender, education and age on the level and course of PTS, in survivors from a non-fatal airliner emergency landing caused by engine failure at an altitude of 1 km. There were 129 persons on board. A survey including the Impact of Event Scale was distributed to 106 subjects after 1 month, 4 months, 14 months, and 25 months, and to 95 subjects after 19 years (response rates 64–83%). There were initially high levels of PTS. The majority of changes in PTS occurred from 1 to 4 months after the event. There were small changes from 4 to 25 months but further decrease in PTS thereafter. Female gender was associated with higher levels of PTS whereas gender was unrelated to the slope of the short- and long-term trajectories. Higher education was related to a quicker recovery although not to initial or long-term PTS. Age was not associated with PTS. The present findings suggest that a life-threatening experience without collateral stressors may produce high levels of acute posttraumatic stress, yet with a benign prognosis. The findings further implicate that gender is unrelated to trajectories of recovery in the context of highly similar exposure and few collateral stressors.

## Introduction

During the last decades there have been major advances in our understanding of psychological consequences of potentially traumatic events. The progress notwithstanding, research on the long-term psychological consequences of single potentially traumatic events has only begun to accumulate. In addition, variations in exposure severity (e.g., proximity to event, experiencing life threat, physical injuries, or traumatic bereavement) present challenges for investigators when trying to evaluate the psychological consequences of a potentially traumatic event.

Survivors often experience a range of distressing psychological reactions and behavioural responses in the immediate aftermath and draws on personal and societal resources to adapt. One common set of reactions is labelled posttraumatic stress (PTS), which include intrusive recollections, disabling avoidance behaviours, dysphoria, and hyperarousal. The recovery process is dynamic and should not be seen as pathological unless prolonged or disruptive of usual functioning [1]. The diagnosis of posttraumatic stress disorder (PTSD) is characterised by the above reactions with the additional criteria that the symptoms must last for ≥1 month and they should lead to a significant functional impairment [2]. A substantial minority of survivors suffer from PTSD even after transient events such as motor vehicle accidents (MVAs). In a study of attenders at a emergency department following a road traffic accident, 20% of the participants with minor or no injuries fulfilled criteria for PTSD at three months, 14% at one year, and 11% three years after their accident [3,4].

Studies from various events show that a few linear trajectories capture the variability in PTS during the first years: resistance, resilience/recovery, and chronic dysfunction [5,6]. A majority of individuals show levels of PTS that fit the resistance or resilience/recovery trajectories. The resistant trajectory is associated with low level of PTS at any given time after the event whereas resilience/recovery is characterized by initial PTS that dissipates reliably during the following months. The chronic trajectory includes a minority of individuals and is characterized by high levels of initial PTS that do not fade within the first years. There is also evidence of a delayed trajectory, where high levels of PTS develop not until after the first six months.

Not as much is known about the course of PTS in a long-term perspective, although the literature clearly suggests that posttraumatic stress reactions become more fixed with the passage of time [7,8]. One early study on MVA survivors found unchanged prevalence rates of PTSD from 1 to 5 years after the event [9]. However, long-term PTSD was linked to having experienced additional accidents. Indeed, one study found that 10% of the participants experienced another accident during 1 to 3 years after the index MVA [4]. A recent prospective study from three to eight years after the World Trade Center terrorist attack found trajectories that were characterized by less variation than previously found [10]. Most studies, however, are retrospective and thus uncertainties remain due to methodological limitations (e.g., recall bias).

As for studies that include both short-term and long-term prospective assessments, they mainly concern harrowing events that are followed by an array of secondary stressors: the existing long-term studies concern events that resulted in a large numbers of deaths [11,12], physical injuries [13], or severe disruption of the community [14], which altogether restricts any generalisations to be made regarding the course of PTS after a transient event. In these studies, approximately one fifth to one fourth of the survivors experienced significant chronic posttraumatic stress or fulfilled criteria for PTSD after more than a decade after the event [11,12,15]. Two studies found that, after an initial decrease from very high levels to moderate levels of PTS, little or no improvement in average posttraumatic stress levels was seen after the first year [11,12], whereas a study of the survivors of a flooding in Buffalo Creek found a decrease from the second to fourteenth year post-event [15].

There are several predictors for PTS, including low education, younger age, and gender [16]. Little is known about how predictors operate on PTS with regard to changes over time. For example, it is unclear if the increased risk of PTS in women is mainly determined by factors that operate during the pre-, peri-, or posttraumatic period. There are mixed findings, with various studies indicating that variations in peritraumatic dissociation, social support resources, and coping processes contribute towards the gender difference [17]. If the gender difference is influenced by differences in coping and social resources then gender-specific trajectories would differ in slope, whereas pre- or peri-traumatic determinants (e.g., peritraumatic dissociation) would be suggested by gender differences in the initial PTS levels. In their study of the Buffalo Creek survivors [15], the authors report that initially, women had poorer mental health than men whereas the symptom burden for men and women were nearly identical after 14 years, suggesting that the changes for women were more pronounced than for men. Similarly, a study of survivors from severe MVAs found higher rates of PTSD among women than men at 1 month but not 6–12 months after the event [18,19].

An imminent threat to physical integrity is the prototypical feature of a potentially traumatic event [2]. Due to the nearly ubiquitous nature of collateral stressors following traumatic events (e.g., bereavement, physical injury, as well as social, practical, and financial issues) [20], little is known about the capacity of a threat to life alone to produce posttraumatic stress, although attempts have been made to disentangle life threat from other experiences during traumatic events [21,22]. Importantly, the variation in severity of exposure to potentially traumatizing material may shroud systematic differences in responses to different events both within samples [21] and across various types of potentially traumatic events [23].

With regard to exposure severity, an airliner emergency landing from high altitude includes several noteworthy features. The life threat is unambiguous, and the external environment and the unfolding of the incident are highly similar for all passengers. They may also be associated with relatively minor financial and material losses. An important characteristic of incidents concerning public transportation is that they are generally centrifugal events in that they occur to temporarily congregated groups of people and distant from the survivors’ communities [24]. Unlike centripetal events, where the whole community is afflicted, survivors from a centrifugal event thus return to more or less intact support systems, an important factor in the process of recovery [25].

Few studies of survivors from aviation disasters have been conducted, partly because of the high mortality rate in the events. The extant research comprises small studies of events associated with severe injuries and fatalities. In a small study, Birmes et al. [26] found that three of eight survivors developed PTSD during the first month after a plane crash in which 23 died and 29 were seriously injured. The participants’ average score on the Impact of Event Scale (IES) was 33. Gregg et al. [27] conducted clinical interviews within one year of the Kegworth air disaster in which 47 people died and nearly all aboard were injured. The PTSD rate was 40% in the first year, and the average IES score was approximately 26. Oe et al. [28] assessed survivors at 6 months, 1 year, and 10 years after an aviation disaster and found that approximately one-fourth of the 26 respondents experienced event-related distress in the long term. To our knowledge, one report exists from a non-fatal event: Sloan [29] studied 30 young men for 12 months after an airplane crash-landing. After two weeks 54% met the symptom criteria for PTSD and the participants had an average IES score of 31. The greatest decrease in reactions occurred during the first 8 weeks with no subsequent changes to the last follow up after 12 months.

In summary, the course of PTS can be characterized as dynamic within the first months or year, while there seems to be less change in the overall burden in the long term. The inherent characteristics of potentially traumatic events limit our knowledge about the psychological reactions to a life-threat itself, and how various predictors influence these reactions. In addition, there is a scarcity of prospective studies on whether and how these reactions persist in a longer perspective.

### Aims

This study reports event-related distress from a non-fatal airliner crash-landing. The primary aim of this study was to assess the course of posttraumatic stress reactions. We expected that there would be a significant change in stress reactions during the first months whereas small changes would be observed in the long-term. The effects of gender, educational attainment, and age on posttraumatic stress levels and trajectories were assessed. Female gender and lower education were expected to be related to higher levels of posttraumatic stress, whereas no directional hypothesis was warranted for age. Ancillary analyses were conducted to assess the role of subsequent adverse life events and treatment on long-term distress.

## Methods

### The Event

Shortly after an airliner (a DC 9–81) took off at 8:48 local time on a December morning, ice broke off from the wings and caused both engines to fail within 78 seconds into the flight. The aircraft rapidly descended without engine power from an altitude of 1,011 m (3,318 ft). Four minutes after takeoff the plane crashed onto a field, with a speed of 107 knots (198 km/h) when hitting the ground. The aircraft slid 110 m (361 ft) and the aircraft body ruptured laterally into three sections. No fire broke out and the aircraft was evacuated swiftly and apparently without panic. Emergency services arrived after 31 minutes. Everyone on board survived. Eleven survivors received hospital care (all but one passenger were discharged within one week) and 13 received outpatient care. Sixty survivors sustained minor fractures, lacerations, and contusions and were not subjected to hospital care. Thirty-nine survivors were uninjured [30].

### Procedure

The survivors filled in a measure of posttraumatic stress within the first two years after the event: after 1 month (T1), 4 months (T2), 14 months (T3), and 25 months (T4) [31]. After 19 years (T5), a postal survey was sent to the survivors for whom addresses could be retrieved, followed by a reminder.

### Participants

There were 123 passengers and 6 crewmembers onboard the aircraft. In the first four surveys, addresses to 106 survivors could be retrieved and the response rates were as follows: 88 (83%) responded at T1; 68 (64%) at T2; 75 (71%) at T3; and 75 (71%) also at T4. After 19 years, 9 survivors were deceased and 22 could not be traced. Thus, 95 surveys were sent out after 19 years and, after one reminder, 70 participants (74%) responded.

### Measures

The Impact of Event Scale (IES) [32] is a widely used questionnaire for self-rated posttraumatic stress and was used in T1–T4. Respondents indicate the frequency of 8 avoidance and 7 intrusion reactions during the past week pertaining to a specific event. The responses are scored 0 (*never*), 1, 3, or 5 (*always*) and a total score is achieved by summing all items (range 0–75). The Impact of Event Scale–Revised (IES-R) [33,34], in which the IES was supplemented with hyperarousal items [35], was used in T5. This version of the IES-R retained the scoring from the IES and comprises a summated score of 22 items (range = 0–110). The IES score was calculated from the IES-R at T5. The Swedish version performed best as a screening measure for PTSD with a cut-off score of 25 (discriminant ability [DA] = 0.83) [35], which was chosen as a cut-off for significant posttraumatic stress in the present study. To include hyperarousal reactions, the proportion of significant posttraumatic stress according to the IES-R was also calculated with a cut-off score of 40 (DA = 0.85) [35]. The IES and the IES-R had a high internal consistency (in the 19-year survey, Cronbach’s α = .90 and. 93, respectively).

The 12-item General Health Questionnaire (GHQ-12) [36] was used to assess general mental health at T5. The GHQ-12 focuses on the inability to undertake normal functions and the appearance of new and distressing phenomena, is sensitive to short-term disorders but not enduring attributes of the respondent [37]. The GHQ-12 is reliable and valid in community samples in different cultural contexts [38]. A sum score of the Likert-coded items (0–1–2–3) was used for the calculation of mean values (range 0–36). The GHQ-scoring method with a cut-off at ≥ 3 points was used to estimate the proportion of participants with a high likelihood of poor mental health [37]. Cronbach’s α = .86 for the summated score.

Adverse life events were assessed after 19 years by a 13-item inventory previously used in a similar survey [11]. The participants were asked to indicate if (*yes*/*no*) and when (*year*) they had experienced any of the following: disaster, war/terror, death of a family member or close friend, threat to physical/psychological integrity, serious disease or injury to self or family member, accident, divorce, or serious financial problems. Participants were then asked to rate the impact of each event on a scale from 1 to 4 (*none*, *small*, *moderate*, or *great*). A total score was achieved by assigning a score of 0 to the events that the participants had not experienced and summing the impact of all indicated events indicated to have occurred after the airliner emergency (range = 0–52).

Perceptions of social support were assessed retrospectively at T5 with the Crisis Support Scale (CSS) [39,40]. The CSS assesses perceived support from friends and relatives, and the respondent is asked to consider support that concerns a specific event. The CSS is composed of six items that are rated on a seven-point scale ranging from *never* (1) to *always* (7). The items assess the degree to which the respondent have perceived (1) *availability of others*, (2) *contact with others with similar experiences*, (3) *possibility to confide in others*, (4) *emotional support*, (5) *tangible support* and (6) *having been let down by others*. A total score was achieved by summing the items, with negative response reversed (range = 6–42).

### Statistical Analysis

At T1–T4 three IES items had missing values from four participants. At T5, five participants had one missing value each on the IES-R. The missing values were imputed by means of the EM procedure in SPSS, using all other IES-R items from the same assessment as predictors. The proportions of participants with significant posttraumatic stress are presented with 95% Clopper-Pearson confidence intervals. Bivariate correlations for IES total and subscale scores at all assessments, including the IES-R hyperarousal subscale at T5, were calculated. As the data set was unbalanced, Generalized Estimating Equations (GEE) [41] was used to assess differences in the levels and course of posttraumatic stress related to gender (men/women), education (university degree or not), and age. GEE uses all available observations instead of using only participants with complete data for all time points. A negative binomial with a log link function was employed, and a first-order autoregressive correlation matrix was defined. The model was refined by the use of linear splines [42], which allows for segmentation of the course of reactions across assessments into sequential linear trends. The segments are joined at fixed times whereas the slopes of the segments are free to vary. The use of splines made it possible to assess the short- and long-term changes separately. The knot was set at T2 according to previous findings [29]. Pairwise *t* tests were used to assess changes in posttraumatic stress from the previous assessment. All tests were two-tailed with α = .05. Data analysis was performed in SPSS version 19 for Mac (SPSS Inc.) except for the GEE analysis, which was performed in SAS software v9 for Windows.

### Ethics Statement

The study protocol was approved by the Regional Ethical Review Board in Uppsala, Sweden. All participants provided written informed consent.

## Results

### Descriptive Analysis

At T5 the participants were on average 53 years of age (*SD* = 11.3). A majority of participants had completed higher education at college or university (*n* = 40; 57%) and many were currently employed or had retired from work (*n* = 59; 84%). The participants were at T5 mostly married or cohabiting (*n* = 45; 64%) while 14 (20%) identified themselves as divorced and not in a relationship, 8 (11%) as singles, and 3 (4%) as widow/widower not in a relationship. Overall, they perceived that they had received a fair amount support related to the event from their friends and relatives (CSS *M* = 30.6; *SD* = 6.9), with 82% of participants responding positively (i.e., scoring ≥ 4) to all CSS items.

One third of the participants reported that, since the emergency landing, they had experienced one or more episodes when they could not function at work or home (*n* = 24; 34%). Thirteen participants indicated that it was a consequence of the emergency landing. During the 19 years since the incident, 28 participants (40%) had experienced no adverse life events that they considered to have a moderate or large impact on their lives when they occurred, while 17 had experienced one, 16 had experienced two, 6 had experienced three, and 3 had experienced four or five events. The most common events were the death of a parent (*n* = 22), divorce (*n* = 18), and the death of a relative/close friend (*n* = 16).

### Posttraumatic Stress

There were 24 participants who responded to all surveys. For these, the IES means (*SD*) were as follows: *M*_T1_ = 29.6 (14.6), *M*_T2_ = 19.5 (14.6), *M*_T3_ = 19.8 (16.2), *M*_T4_ = 24.4 (20.8), and *M*_T5_ = 14.8 (14.0), all highly similar to the means for the total sample (Table 1). According to the IES-R, there were 11 participants (16%) with significant posttraumatic stress after 19 years, 95% CI [8.6%, 25.7%]. Table 2 shows the bivariate correlations among IES total scores as well as with the hyperarousal subscale that was administered only at T5. For comparison purposes, participants with complete data were included. There were strong associations among the IES scores at different assessments. The associations evidenced an autoregressive pattern and were generally somewhat weaker when involving T1 as compared to associations among the T2 to T5 scores.

**Table 1:** Posttraumatic stress in survivors from an airliner emergency landing.

**Time from event**	***N* (women)**	**Age at event**	Impact of Event Scale		***t*-value (*df*)^b^**
		***M* (*SD*)**	***M* (*SD*)**	**Cases^a^ [95% CI]**	
**1 month**	88 (31%)	34 (12)	28.1 (15.7)	59% [48, 70]	
**4 months**	68 (50%)	36 (12)	20.2 (14.8)	35% [24, 48]	5.30 (46)***
**14 months**	75 (51%)	35 (12)	23.5 (17.9)	35% [24, 47]	–1.01 (59)
**25 months**	76 (49%)	36 (12)	25.1 (20.5)	41% [30, 53]	–1.74 (59)
**19 years**	70 (43%)	35 (11)	14.1 (13.1)	20% [11, 31]	4.25 (51)***

^a^Indicates proportion of participants with significant posttraumatic stress (≥ 25 cut-off score).

^b^Indicates change in average posttraumatic stress from the previous assessment based on pairwise comparisons.

*** *p* < .001

**Table 2:** Correlations among assessments of posttraumatic stress in survivors from an airliner emergency landing.

**Assessment**	**T2**	**T3**	**T4**	**T5**	**Hyperarousal^a^ T5**
**T1: 1 month**	.58	.51	.53	.44	.38
**T2: 4 months**	—	.77	.72	.68	.51
**T3: 14 months**		—	.75	.71	.51
**T4: 25 months**			—	.82	.72
**T5: 19 years**				—	.82

For comparison purposes, only participants who responded to all surveys were included (*n* = 24). Correlations greater than ±0.40 are significant at *p* < .05 (2-tailed) and correlations greater than ±0.51 are significant at *p* < .01 (2-tailed).

^a^The Impact of Event Scale–Revised, in which hyperarousal items are included, was administered only at 19 years.

**Fig 1:**
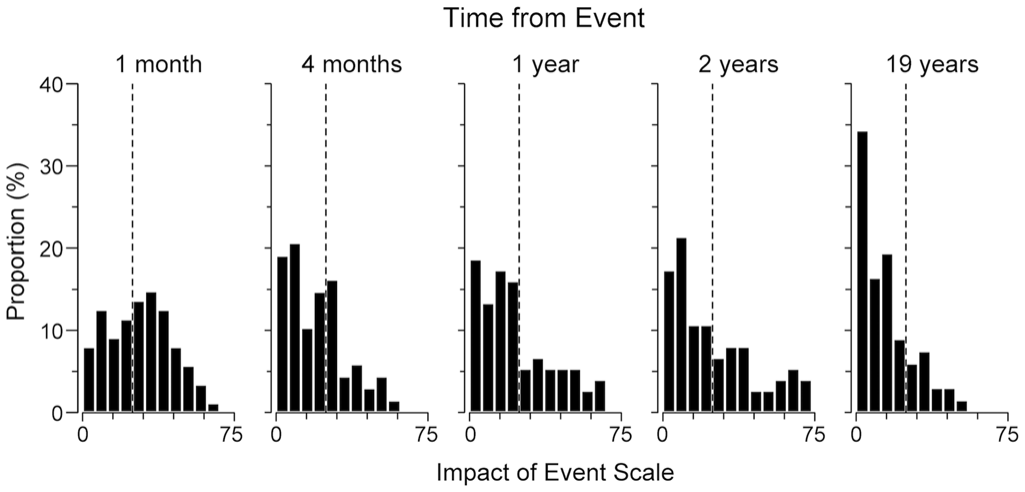
Posttraumatic Stress in Surviving Passengers from an Airliner Emergency Landing. The dashed line denotes the cut-off score for significant posttraumatic stress (total score ≥ 25).

In a GEE analysis that regressed IES scores on time, as defined by the two linear splines, the estimated average level of posttraumatic stress changed from T1 to T2 by -24%, 95% CI [-31, -15], Wald χ^2^ = 23.88, *p* < .001. The annual rate of change from T2 to T5 was estimated to –2.7% [–3.8, –1.6], amounting to a total of –41%, Wald χ^2^ = 23.38, *p* < .001. In total, the estimated change from T1 to T5 was –54%. The model fit reasonably well compared with the actual IES scores. However, the actual scores increased somewhat from T2 to T4. For a more detailed analysis, pairwise *t* tests were used to compare the change in IES from each previous assessment. As seen in Table 1, only the T1–T2 and the T4–T5 changes were statistically significant. The distributions of IES scores across assessments are illustrated in Fig. 1.

**Fig 2:**
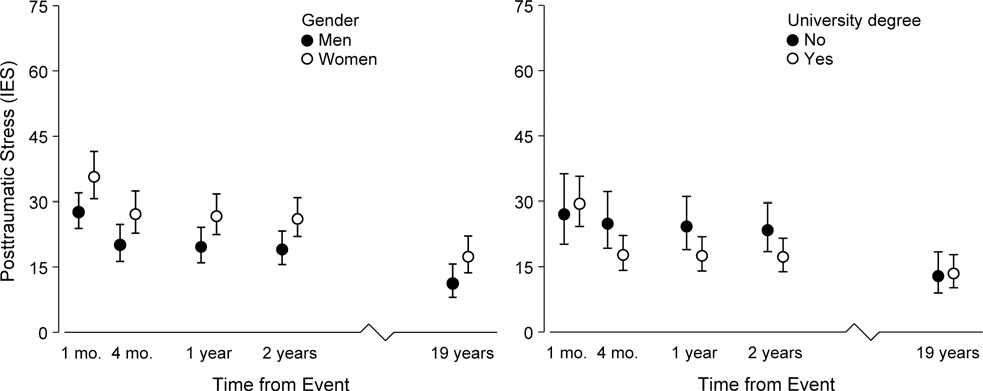
Estimated Mean Levels with 95% Confidence Intervals of Posttraumatic Stress as Assessed by the Impact of Event Scale (IES) when Regressed on Gender and Education in Survivors from an Airliner Emergency Landing.

When gender was entered into the GEE model (see Table 1 for the gender distribution at each assessment) the average posttraumatic stress level at T1 for men were estimated to be lower than for women, est. -22%, 95% CI [-4, -37], Wald χ^2^ = 5.86, *p* = .018. The interaction terms for time and gender did not show any differences due to gender in the short- or long-term trajectories of posttraumatic stress: T1–T2 × Gender, Wald χ^2^ = 0.185, *p* = .68, and T2–T5 × Gender, Wald χ^2^ = 0.169, *p* = .68. The essentially parallel trajectories yielded an estimated T5 score that was lower for men than for women (-32%; *M*_diff_ = -6.1, *SE*_diff_ = 2.87), Wald χ^2^ = 4.31, *p* = .038 (Fig. 2). In the GEE model with educational attainment as a predictor, education was not related to posttraumatic stress at 1 month, Wald χ^2^ = 0.218, *p* = .64. However, higher education was related to a steeper trajectory in the short term, T1–T2 × Education Wald χ^2^ = 7.74, *p* = .0054. The difference resulted in an estimated T2 score that was marginally lower for those with a higher education as compared to those with a lower education, est. –29%, 95% CI [0.001, -49], Wald χ^2^ = 3.83, *p* = .050. Higher education was related to less change in the long term, T2–T5 × Education Wald χ^2^ = 7.82, *p* = .0052, such that at T5 there were no difference related to education, Wald χ^2^ = 0.04, *p* = .84 (Fig. 2). In a GEE model with age as a predictor, age was not found to be associated with the level of posttraumatic stress, Wald χ^2^ = 3.22, *p* = .07, or with the rate of change in the short-term, Wald χ^2^ = 0.008, *p* = .90, or in the long-term, Wald χ^2^ = 0.001, *p* = .90. Finally, in a combined analysis including the main effects of gender and education, their interaction, and the significant interactions between education and short- and long-term changes, the Gender × Education interaction was not significant, Wald χ^2^ = 1.52, *p* = .22. The other parameters were highly similar to those reported above.

### Ancillary Analyses

There were 11 participants with poor general mental health according to the GHQ-12. Only one participant had both significant posttraumatic stress and poor general mental health and there was a small correlation between posttraumatic stress and poor general mental health (GHQ-12 sum score), *r*(68) = .098, *p = *.42.

The posttraumatic stress scores may have been influenced by adverse life events that occurred after the emergency landing in 1991. However, no association was found between the impact of adverse life events after 1991 and posttraumatic stress at any timepoint, *r*s = -.001 to .15, *p*s = .99 to .19. A moderate association was found between life events and poor general mental health as assessed at T5 by the GHQ-12, *r*(57) * = * .258, *p* = .031.

There were 24 (34%) participants who indicated that they had received treatment for mental health concerns. Thirteen participants (19%) had received treatment for severe stress reactions, 16 (23%) for sadness or depression, 14 (20%) for worry or anxiety, 8 (11%) for sleeping difficulties, and 2 (3%) for other conditions. Nine participants had received psychological treatment, 4 had received pharmacological treatment, and 11 had received both. The median overall time in treatment was approximately two years for both psycho- and pharmacotherapy (range = 2 weeks to 17 years). The median treatment length for severe stress reactions was 1.5 years. The participants who had received treatment for severe stress reactions had higher scores on IES-R after 19 years than those who had not received treatment for severe stress (*M*_diff_ = 16.6, *SE*_diff_ = 5.4), *t*(68) = 3.04, *p* = .003, Hedges’ *g* = 0.92. However, the participants who had received treatment had higher scores on the IES at all previous assessments. For T1 (*M*_diff_ = 18.5, *SE*_diff_ = 4.8), *t*(68) = 3.84, *p* < .001, *g* = 1.41. The differences at T2–T4 (data not shown) were similar to the difference at T1.

## Discussion

The present findings demonstrate that PTS can develop, and in some cases persist for many years, in survivors from a single non-fatal airliner emergency landing. The average level of PTS decreased in two waves, initially during 1–4 months and then during 2–19 years after. As expected, women experienced higher levels of PTS than men whereas the trajectories of stress reactions were not related to gender. In contrast, higher education was related to a greater reduction in PTS during the first months, which was offset in the long term such that education was not related to posttraumatic stress at 19 years.

The initial levels of PTS were similar to previous studies of initial stress reactions after more severe aviation disasters [26–28]. The present findings further indicate that there were nominal yet statistically not significant increases in PTS from 4 months to 2 years after. This raises concerns with regard to the similarity of the samples among assessments. However, the comparison of respondents and nonrespondents showed a high similarity in PTS severity and the composition of the sample was similar across assessments. In addition, the initial trajectory of PTS was very similar to the findings by Sloan [29]. The present study extended the findings by Sloan in that after a period of little or no change a subsequent decrease seem to occur. The point estimate of significant PTS after 19 years, 16% according to the IES-R and 20% according to the IES, indicate a somewhat lower proportion of long-term PTS compared with other long-term studies [11,12]. However, the differences are small and the wide confidence intervals of the estimates overlap.

There were strong associations between IES scores at T2–T4 and T5, and the pattern of associations indicated that the autoregressive correlations. Strong associations between initial and very long-term assessments of PTS have been reported in a study of a ferry disaster [11]: after that event, the correlations between early IES scores (3 months to 3 years after) and IES scores at 14 years was *r* = .59 to. 80. These findings corroborate the notion of between-subject stability in PTS in a very long-term perspective, although in the context of declining overall levels within subjects.

No differences could be detected between men and women in short- or long-term trajectories of PTS, suggesting that the greater burden of PTS in women than men is owing to initially stronger reactions not offset by a greater reduction later on. These findings are consistent with the literature, which generally point to pre-event (e.g., biological differences) and peri-event factors (e.g., dissociative responses, peri-traumatic emotionality) as putative mechanisms for the gender differences in PTS [17,43,44]. The present findings further suggest that the size of this difference is stable across many years, as seen in the context of nearly identical exposure among survivors and few secondary stressors. Although speculative, it seems that the studies that have found gender differences in the trajectories of PTS were concerned with severe events entailing several and prolonged secondary stressors, such as in the Buffalo Creek study [15].

Higher education was related to a steeper decline in PTS but not to severity at 1 month or 19 years. Lower education has in several studies been linked to increased levels of chronic PTS [16]. It is reasonable to believe that educational attainment acts as a buffer through functions related to individual and social resources, and as such potentially has greater effects in the long term after events with greater and more long-standing stressors (i.e., disrupts these functions).

There are a number of limitations to keep in mind when drawing conclusions from this study. First, the small sample size yields imprecise point estimates, particularly for the sub-group analyses, and may inflate prevalence rates. Another limitation is the reliance on self-report measures. However, the IES is perhaps the most widely used questionnaire for the assessment of PTS and its validity and reliability have been established in several studies [45]. Nonetheless, the hiatus of 17 years until the 19-year survey gave ample time for maturation in subjects or other factors that may have caused bias in the comparison of short- and long-term distress; these issues are compounded by the lack of a comparison group.

Finally, we note that the participants who reported high levels of PTS did not report poor general mental health. One reason for this may be that the GHQ-12 focuses on the appearance of new and distressing phenomena [37] whereas the PTS reactions had been present for several years. The association between PTS and general mental health has been found to be greater in long-term studies after more severe events [11,12]. For example, Bøe et al. found that general psychopathology 27 years after a severe oil platform disaster was associated with disaster exposure [12]. The discrepancy between IES and GHQ points to the possibility that long-term PTS from a circumscribed life-threatening event with few collateral stressors is not associated with impairments in functioning. Rather, the PTS reactions that remain after 19 years may represent side effects of adaptation [46].

### Conclusions

The present findings suggest that the experience of a transient yet very real threat to life and almost none collateral stressors can lead to high levels of acute PTS, as high as in samples of survivors from devastating disasters. The benign circumstances surrounding the present event, however, seem to have conferred chances for a rapid diminution of PTS reactions, and for low levels in a long-term perspective. Long-term stress reactions are likely to be minor and seem unrelated to the survivors’ experience of more transient episodes of poor mental health, suggesting that very long-term PTS may come with adaptation: this is an interesting area for further study. Further investigations using measures of distress and diagnostic interviews may clarify whether the reactions associated with the event are distressing or debilitating in the long term.

In the context of nearly identical exposure to life threat among the survivors, and few secondary stressors, the gender differences in the levels but not in the slopes of PTS lend support to such differences being attributed to pre- or peri-traumatic factors. Further studies may want to investigate more closely whether gender differences in posttraumatic stress trajectories may be related to the characteristics of the post-event context. In contrast, the different slopes in education show how putative post-traumatic factors, such as individual and social resources, influence the speed of adaptation but not necessarily the long-term outcome. These findings underscore the importance of longitudinal, long-term studies to capture processes in coping with extreme stress.
